# High‐frequency peripheral vibration decreases completion time on a number of motor tasks

**DOI:** 10.1111/ejn.14050

**Published:** 2018-08-06

**Authors:** Antonella Macerollo, Clare Palmer, Thomas Foltynie, Prasad Korlipara, Patricia Limousin, Mark Edwards, James M. Kilner

**Affiliations:** ^1^ Sobell Department of Motor Neuroscience and Movement Disorders UCL London UK

**Keywords:** bradykinesia, motor performance, Parkinson's disease, vibration

## Abstract

A recent theoretical account of motor control proposes that modulation of afferent information plays a role in affecting how readily we can move. Increasing the estimate of uncertainty surrounding the afferent input is a necessary step in being able to move. It has been proposed that an inability to modulate the gain of this sensory information underlies the cardinal symptoms of Parkinson's disease (PD). We aimed to test this theory by modulating the uncertainty of the proprioceptive signal using high‐frequency peripheral vibration, to determine the subsequent effect on motor performance. We investigated if this peripheral stimulus might modulate oscillatory activity over the sensorimotor cortex in order to understand the mechanism by which peripheral vibration can change motor performance. We found that 80 Hz peripheral vibration applied to the right wrist of a total of 54 healthy human participants reproducibly improved performance across four separate randomised experiments on a number of motor control tasks (nine‐hole peg task, box and block test, reaction time task and finger tapping). Improved performance on all motor tasks (except the amplitude of finger tapping) was also seen for a sample of 18PD patients ON medication. EEG data investigating the effect of vibration on oscillatory activity revealed a significant decrease in beta power (15–30 Hz) over the contralateral sensorimotor cortex at the onset and offset of 80 Hz vibration. This finding is consistent with a novel theoretical account of motor initiation, namely that modulating uncertainty of the proprioceptive afferent signal improves motor performance potentially by gating the incoming sensory signal and allowing for top‐down proprioceptive predictions.

## 1 INTRODUCTION

Every movement we make stimulates peripheral sensory receptors that provide sensory feedback of the motor act. Influential models of motor control have proposed that when we move we predict the sensory consequences of that movement (through forward models) and compare this prediction to the actual sensory input (Adams, Shipp, & Friston, 2013; Wolpert & Ghahramani, 2000). Any difference between the predicted and actual sensory input results in a prediction error, which is used to update the forward model for more accurate future predictions. In order to determine the relevance of any prediction errors, the model requires estimates of both the uncertainty in the motor prediction and the uncertainty of the actual sensory input (Körding & Wolpert, 2004). The importance of the estimate of uncertainty at both of these levels is highlighted in a recent theoretical account of motor control and movement initiation: active inference (Friston, Mattout, & Kilner, 2011). Within this framework increasing the estimate of the uncertainty surrounding the afferent input leads to an attenuation of the sensory signal, which is a necessary step in order to move. It has been proposed that an inability to modulate the gain of this sensory information underlies one of the cardinal symptoms of Parkinson's disease (PD): bradykinesia, the slowness of movement (Palmer, Zapparoli, & Kilner, 2016). Indeed, PD patients show a deficit in the gating of somatosensory evoked potentials (SEPs; Macerollo et al., 2016), which are known to be attenuated with movement (Starr & Cohen, 1985). Here, we aimed to test one prediction of this theory by modulating the uncertainty of the proprioceptive signal, using high‐frequency peripheral vibration, to determine the subsequent effect on motor control.

It has previously been shown that high‐frequency vibration of forearm muscle tendons, which selectively activates muscle spindles (Brown, Engberg, & Matthews, 1967; Burke, Hagbarth, Löfstedt, & Wallin, 1976; Roll, Vedel, & Ribot, 1989), produces the illusion that the arm is moving or has been displaced in the absence of any EMG activity (Craske, 1977; Goodwin, McCloskey, & Matthews, 1972; McCloskey, 1973). This illusion reflects the central nervous system incorrectly interpreting the increased firing rate of muscle spindles as if the vibrated muscle is lengthening; this generates uncertainty in the actual position of the limb. This has been demonstrated in a number of position‐matching and pointing tasks all of which show increased error, or reduced accuracy, following high‐frequency peripheral vibration (Capaday & Cooke, 1983; Cordo, Gurfinkel, Bevan, & Kerr, 1995; Cordo, Gurfinkel, Brumagne, & Flores‐Vieira, 2005; Inglis & Frank, 1990; Tsay, Giummarra, Allen, & Proske, 2016). Importantly, prolonged high amplitude vibration (30–100 Hz) can produce an increase in muscle contraction referred to as the tonic vibration reflex (TVR). This reflects an increase in activity from afferent nerve fibres that activate monosynaptic and polysynaptic reflex arcs (Eklund and Hagbarth, 1966). As in these previous experiments, in the current study, low amplitude vibration was employed to activate muscle spindles without any overt muscle contraction. In this study we sought to understand how this interruption to normal proprioceptive processing could influence movement initiation. We hypothesised that increasing proprioceptive uncertainty by giving high‐frequency peripheral vibration prior to movement would improve motor initiation in both healthy subjects and PD patients in line with the theoretical accounts outlined above.

It has been suggested that the modulation of beta power over sensorimotor cortex prior to and during movement may mediate the sensory gating theorised to be necessary for movement (Palmer et al., 2016). We hypothesised that vibration may enhance movement speed by increasing preparatory beta desynchronisation placing the cortex in a more “ready‐to‐move” state. To test this, we measured EEG in a sample of healthy subjects before, during and after peripheral vibration while subjects were at rest.

In summary, the aim of the series of experiments described here was to test the hypothesis that increasing somatosensory afferent uncertainty, using high‐frequency vibration, would lead to a measurable change in simple movements, reflecting faster movement onset and movement initiation. This was tested both in healthy subjects and patients with PD.

## 2 MATERIAL AND METHODS

### 2.1 Behavioural study

#### 2.1.1 Procedure and experimental design

##### Experiment 1

**Figure 1:**
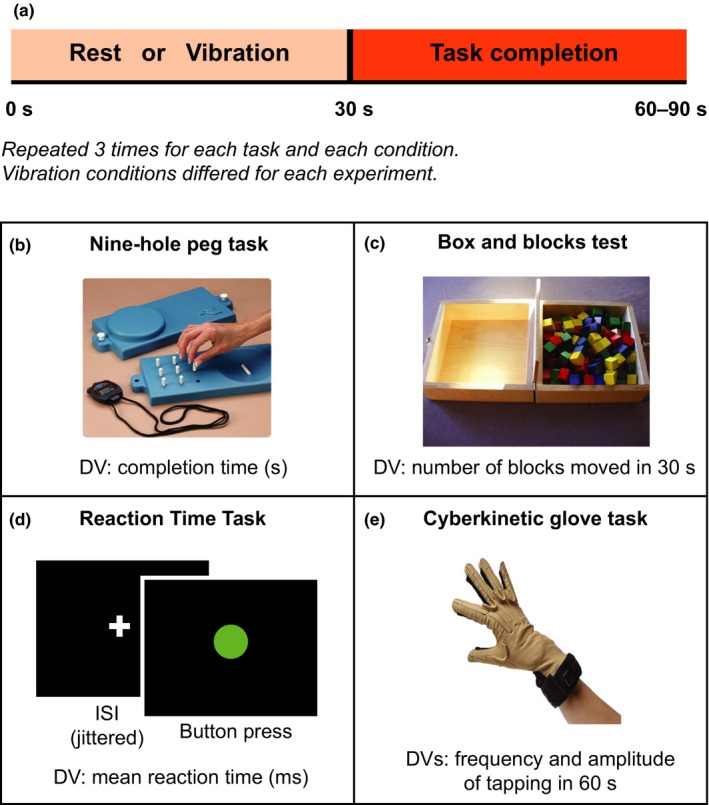
Experimental protocols used throughout the study. (a) In each experiment, 30 s of 80 Hz vibration or no vibration or a control condition was given prior to completing a motor task. We tested the effect of high‐frequency vibration on the completion time of several motor tasks. Each condition (vibration, rest or control) as well as each task was repeated three times. Particularly, Experiment 1 tested two conditions: 30 s vibration at 80 Hz on the right wrist vs. no vibration; Experiment 2 tested three conditions: 30 s vibration at 80 Hz on the right wrist, no vibration and 80 Hz on the left wrist (control condition); Experiment 3 and Experiment 4 (PD patients) tested the following three conditions: 30 s vibration at 80 Hz on the right wrist, no vibration and 20 Hz on the right wrist (control condition). All experiments except Experiment 4 used healthy subjects. In all experiments, the motor performance was measured throughout using the following three tasks: 9 peg hole test (b), box and blocks task (c) and reaction time test (d). In the Experiment 3 as well as 4, we also measured the amplitude and frequency of the tapping with the cyber glove (e). [Colour figure can be viewed at http://wileyonlinelibrary.com]

Eighteen right‐handed healthy participants (nine men, nine women, and mean age 30.5 years, range 19–39 years) underwent the assessment of the motor performance of the right hand through three different tasks: (a) the box and blocks test (Mathiowetz, Volland, Kashman, & Weber, 1985); (b) the nine‐hole peg test (Grice et al., 2003); (c) a reaction time task (custom code written in MATLAB 2015a; Figure 1). For the box and blocks test, subjects were instructed to move as many blocks as they could from one box to another in 30 s. The total number of blocks moved was the dependent variable recorded. For the nine‐hole peg task, subjects were instructed to place nine pegs into the nine holes as quickly as possible. Participants were given three attempts at this and the average time recorded. For the reaction time task, subjects were instructed to look at a central fixation cross on a laptop screen and press the space bar on the keyboard when a green GO signal appeared. The time between the onset of the fixation cross and the green GO signal was either 500, 750 or 1,000 ms and jittered between trials so the onset of the GO signal was not predictable. The mean reaction time over trials was used the dependent variable for this task. Each task was repeated in two different conditions in randomised order: (a) absence of external stimulus; and (b) following 30 s of vibratory stimuli on the right wrist. Vibratory stimuli were delivered via an electromagnetic mechanical stimulator (Ling Dynamics System) with a 3‐cm‐diameter circular probe under the palm wrist of the right hand. The probe was positioned orthogonally to, and under slight pressure, against the wrist of the right hand. The vibration frequency was 80 Hz based on previous research showing that vibration at this frequency drives kinaesthetic illusions and thus modulates proprioceptive uncertainty (Goodwin et al., 1972; McCloskey, 1973). In each condition, the single motor task was repeated three times. The order of conditions was counterbalanced across participants in each group. The study was approved by the local institutional ethics committee, which was the East of Scotland Research Ethics Service (EoSRES). Written informed consent was obtained from all participants.

##### Experiment 2

The 18 healthy participants previously included in the Experiment 1 performed the blocks and box test, the nine‐hole peg test and the reaction time task with the *right hand* in three different conditions in the randomised order: (a) absence of vibratory stimuli; (b) following 30 s of 80 Hz vibration on the right wrist; and (c) following 30 s of 80 Hz vibration on the left wrist. In each condition, the single motor task was repeated three times. The order of conditions was counterbalanced across participants in each group. Written informed consent was obtained from all participants.

##### Experiment 3

Eighteen naïve right‐handed healthy participants (nine men, nine women, and mean age 30.2 years, range 20–40 years) underwent the assessment of the motor performance of the right hand through four different tasks: (a) the box and blocks test; (b) the nine‐hole peg test; (c) a reaction time task; and (d) 1 min right hand tapping test with the cybernetic glove (Figure 1), which recorded the amplitude as well as the frequency of the tapping between the first two fingers of the right hand. Each task was repeated in three different conditions in the randomised order: (a) absence of vibratory stimuli; (b) following 30 s of 80 Hz vibration on the right wrist; and (c) following 30 s of 20 Hz vibration on the right wrist. In each condition, the single motor task was repeated three times. The order of conditions was counterbalanced across participants in each group. Written informed consent was obtained from all participants.

##### Experiment 4

Eighteen patients with idiopathic PD (11 men, seven women, mean age 65.5 years, range 49–78 years, Table 1) were involved in the study. Idiopathic PD was diagnosed according to the UK PD Society Brain Bank criteria (Goetz et al., 2008) and further confirmed by abnormal dopamine transporter (DaT) SPECT in all patients. None of the patients had disabling tremor. None of the participants were on any non‐PD medications that could affect the measurements performed. All participants were right‐handed. Clinical disease severity was assessed with the motor section (items 3.1–3.18) of the Movement Disorder Society–sponsored revision of the Unified Parkinson's Disease Rating Scale (UPDRS; Goetz et al., 2008). Patients were assessed in the ON state 1 hr after taking levodopa and 2 hr of taking dopamine agonists.

**Table 1:** Clinical and demographic characteristics of patients with Parkinson disease

Patients	Age (y)	Gender	Disease duration (y)	UPDRS III (RUL)	Treatments
1	52	M	10	8	LD + D
2	49	M	3	6	LD + D
3	72	F	3	11	D
4	70	M	3	9	D
5	73	M	10	8	LD + D
6	60	F	5	4	LD + D
7	61	F	9	12	D
8	70	F	5	5	LD + D
9	65	F	10	12	D
10	75	M	6	4	LD + D
11	53	M	2	10	D
12	73	M	10	4	LD + D
13	72	M	10	11	D
14	65	M	8	9	LD + D
15	78	M	10	9	D
16	61	M	6	6	LD
17	64	F	6	5	LD + D
18	67	F	9	8	LD + D
Mean ± *SD*	65.5 ± 8.3	F7/M11	6.9 ± 2.9	8.05 ± 2.94	

Note

D: Dopaminagonist; LD: Levodopa; RUL: right upper limb; *SD*: standard deviation; UPDRS: Unified Parkinson's Disease Rating Scale; y: years.

The 18 PD patients were investigated with the same protocol of Experiment 3. Written informed consent was obtained from all participants.

#### 2.1.2 Data analysis

We made measurements of the following parameters:

- Box and blocks test: mean number of cubes moved from one box to the other box in 30 s.
- Nine‐hole peg test: mean completion time of the test
- Reaction time test: mean reaction time.
- Tapping test with the cyber glove: mean frequency and amplitude of the tapping over 1 min of time window.

These were the measures of movement performance in our study. Firstly, we calculated the mean value as well as the corrected mean value for each parameter. The mean‐corrected values removed the between subject effect by removing the mean value across conditions for each subject.

The Experiment 1 included only two conditions (80 Hz vibration vs. no vibration). Therefore, we performed a simple *t* test to investigate the impact of our condition.

A repeated measures multiway analysis of variance (ANOVA) was conducted on the data of the other behavioural experiments using the following factors: condition (vibration, no vibration, control condition) and the mean values of the analysed parameter.

Post hoc tests were conducted with Bonferroni corrections for multiple comparisons. *p* Values less than 0.05 were considered to be significant. SPSS Statistics software (version 22.0.0) was used for the statistical analysis of the first two parameters. Matlab software (version 2015a) was used to analyse the reaction time results and the frequency and amplitude of the tapping.

### 2.2 Neurophysiology study

#### 2.2.1 Experiment 5

Eighteen healthy participants (five men, 13 women, and mean age 27.1 years, range 20–44 years) were included in the study. One subject was excluded from subsequent EEG analyses due to noisy EEG data.

##### Procedure and experimental design

The neurophysiological study consisted of electroencephalogram (EEG) recording for 10 blocks of 2‐min duration. The 2‐min interval was divided into the following sections: (a) 30 s without vibratory stimuli; (b) 30 s of econtinuous vibratory stimulus at 80 Hz (five blocks) or 20 Hz (five blocks) frequency applied on the right wrist; and (c) 60 s without vibratory stimuli. The order of the two conditions was counterbalanced across participants. Furthermore, the participants underwent the assessment of motor performance of the right hand through the nine‐hole peg test. The task was repeated in three different conditions: (a) absence of vibratory stimuli; (b) following 30 s of 80 Hz vibration on the right wrist; and (c) following 30 s of 20 Hz vibration on the right wrist. In each condition, the single motor task was repeated three times. The order of conditions was counterbalanced across participants in each group. Written informed consent was obtained from all participants.

##### EEG preprocessing and analysis

Electrical activity was recorded at the scalp using a 128 channels Bio semi ActiveTwo AD‐box EEG. EEG was recorded at 2,048 Hz. Offline the data were epoched to the time of the onset of the vibration taking the 30 s before vibration and 90 s after the onset of vibration. The data were high‐passed filtered at 0.1 Hz, low pass filtered at 100 Hz and downsampled to 400 Hz. Prior to frequency analysis, the data for each of the 10 blocks for both vibration conditions were concatenated into one file. To calculate the power spectra, for each of the 10 blocks, the time series was divided into 5‐s independent windows and the power spectra were calculated over this window with 5‐s nonoverlapping time windows of 400 data points using the Welch's averaged periodgram method. This resulted in a time‐frequency power spectrum with a power spectra every 5 s with 1 Hz resolution. The resulting power spectra were then averaged over blocks, log‐transformed and were baseline corrected by subtracting the mean power in the 15 s before vibration onset for each frequency.

An initial analysis focused on modulations in power in the beta frequency range. To this end for each subject and both conditions for each electrode, the time‐frequency data were averaged over the 15–30 Hz frequency range and converted to an image, creating one scalp map for each of the 24 power spectra over time. These 3‐D images were smoothed and analysed in SPM12. To test for differences between the conditions for each subject the time‐scalp images of the beta power modulation for the 80 Hz vibration was subtracted from the 20 Hz vibration and any difference between the two was tested using the standard mass univariate approach in SPM12. The results of this analysis revealed where on the scalp beta power was modulated during vibration and, from this, electrodes of interest were selected (Figure 1a‐c). Subsequent analyses were conducted on the average time‐frequency images over these electrodes of interest. To test for differences between the conditions for each subject the time‐frequency images for the 80 Hz vibration were subtracted from the 20 Hz vibration and any difference between the two was tested using the standard mass univariate approach in SPM12. In addition, modulations in the 80 Hz vibration condition were compared to the baseline power by testing for differences from 0 for just the 80 Hz condition using the standard mass univariate approach in SPM12. All statistical thresholds were corrected for multiple comparisons using random field theory approach on the peak voxel (Kilner, Mattout, Henson, & Friston, 2005).

## 3 RESULTS

### 3.1 Experiment 1

**Figure 2:**
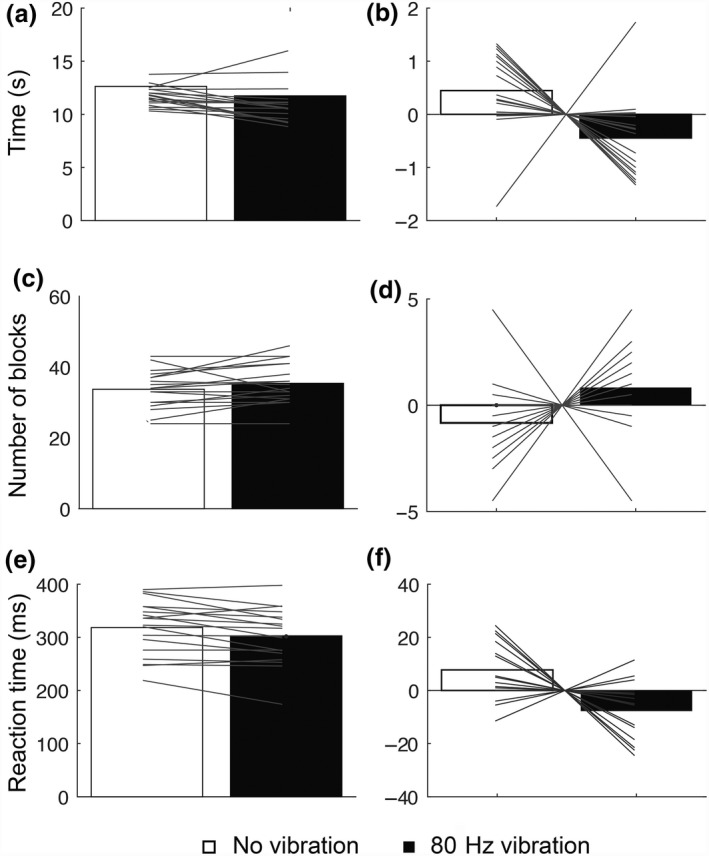
Results of experiment 1. Healthy subjects show improved motor performance following 80 Hz vibration compared to no vibration. Bar graphs show the mean and the corrected mean of completion time for the nine‐hole peg task (a, b), of the number of the blocks moved in 30 s on the blocks and box test (c, d), and of the reaction time task (e, f). 80 Hz vibration (black bars). No vibration (white bars)

There was a significant difference in the mean completion time of the nine‐hole peg test between the two conditions (absence and presence of vibration; *t*_17_ = 2.532, *p* = 0.02). After 30 s of 80 Hz peripheral vibration, the nine‐hole peg task was completed in a faster time (11.73 ± 1.81 s) than after no vibration (12.63 ± 0.89 s; Figure 2a,b). No significant difference was found in the performance of the box and blocks test between the two conditions although the trend was for more boxes to be moved in the same time period following 80 Hz vibration (36 ± 6 boxes) than after no vibration (34 ± 5 boxes), (*t*_17_ = −1.822, *p* = 0.08; Figure 2c,d). The mean reaction time was significantly faster in the reaction time task following 80 Hz vibration (302.83 ± 52.82 ms) than after no vibration (318.33 ± 51.39 ms), (*t*_17_ = 3.046, *p* = 0.007; Figure 2e,f).

### 3.2 Experiment 2

Having demonstrated in Experiment 1 that 30 s of 80 Hz vibration applied to the musculotendinous junction of the right wrist had a significant effect on movement times compared to no vibration, Experiment 2 aimed to introduce a control condition to discount a placebo effect.

**Figure 3:**
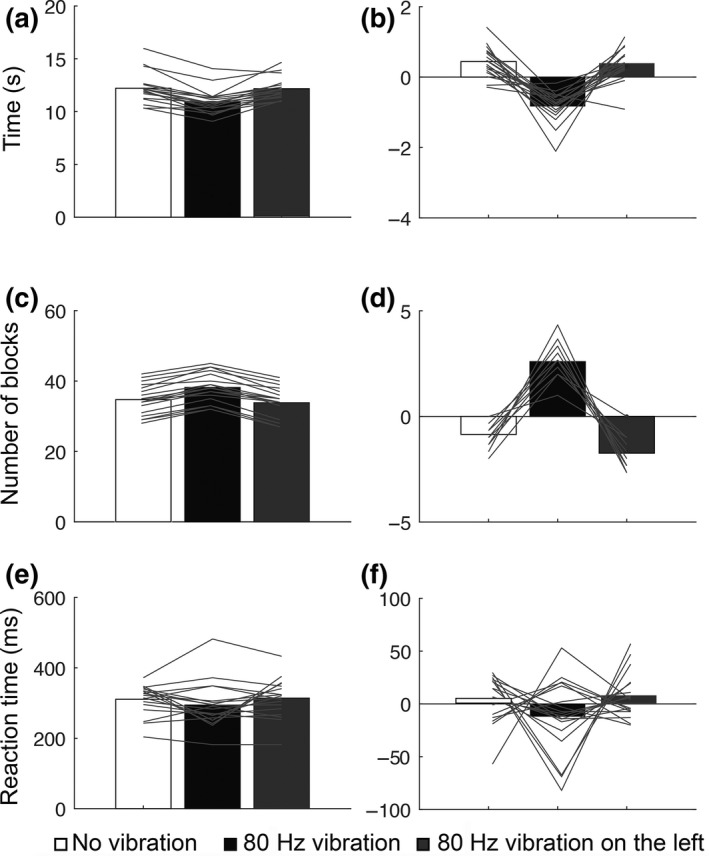
Results of Experiment 2. Healthy subjects show improved motor performance following 80 Hz vibration specifically to the moving hand compared to the nonmoving hand. Bar graphs show the mean and the corrected mean of completion time for the nine‐hole peg task (a, b), of the number of the blocks moved in 30 s on the box and bocks test (c, d), and of the reaction time task (e, f). 80 Hz vibration to the wrist of the moving hand (black bars). 80 Hz vibration to the wrist of the nonmoving hand (grey bars). No vibration (white bars)

A repeated measures ANOVA revealed a significant main effect of condition on the mean completion time of the nine‐hole peg test, (*F*_2,34_ = 31.686, *p* = 0.000, *η* ^2^ = 0.651; Figure 3a,b). Post hoc pairwise comparisons corrected for multiple comparisons revealed a significant difference between mean completion time following 80 Hz vibration on the right wrist (10.94 ± 1.14 s) and no vibration (12.21 ± 1.45 s), (*t*_17_ = 7.351, *p* = 0.000), and between 80 Hz vibration on the right wrist and 80 Hz vibration on the left wrist (12.17 ± 1.03 s), (*t*_17_ = −6.483, *p* = 0.000). There was no significant difference between mean completion time following 80 Hz vibration on the left wrist and no vibration, (*t*_17_ = 0.257, *p* = 0.8).

For the box and blocks test, a repeated measures ANOVA showed a significant main effect of condition on motor performance, (*F*_2,34_ = 116.978, *p* = 0.000, *η* ^2^ = 0.873; Figure 3c,d). Post hoc pairwise comparisons corrected for multiple comparisons revealed a significant difference between the number of cubes moved from one box to the other following 80 Hz vibration on the right wrist (38 ± 4 boxes) and no vibration (35 ± 4 boxes), (*t*_17_ = −11.717, *p* = 0.000), as well as between 80 Hz vibration on the right wrist and 80 Hz vibration on the left wrist (34 ± 5 boxes), (*t*_17_ = 11.985, *p* = 0.000). There was no significant difference between the performance of the test following 80 Hz vibration on the left wrist and no vibration, (*t*_17_ = −0.236, *p* = 0.8). For the reaction time task, there was no significant main effect of condition on mean reaction time (*F*_2,34_ = 1.856, *p* = 0.1, *η* ^2^ = 0.098). There was no significant difference between reaction time following 80 Hz vibration and no vibration (*t*_17_ = 1.3, *p* = 0.2).

There was no significant difference between the mean reaction times following 80 Hz vibration on the right wrist and 80 Hz vibration on the left wrist (*t*_17_ = −1.544, *p* = 0.1) as well as between no vibration and 20 Hz vibration (*t*_17_ = −0.512, *p* = 0.6; Figure 3e,f).

### 3.3 Experiment 3

Having demonstrated in Experiments 1 and 2 that 30 s of 80 Hz vibration applied to the musculotendinous junction of the right wrist had a significant effect on motor performance, Experiment 3 aimed to test whether the frequency of stimulation was critical for the observed modulations in motor performance and in turn provide a more optimal control condition. To this end we investigated the effect of vibration at 80 and 20 Hz to control for any potential placebo effect of vibration at the wrist.

**Figure 4:**
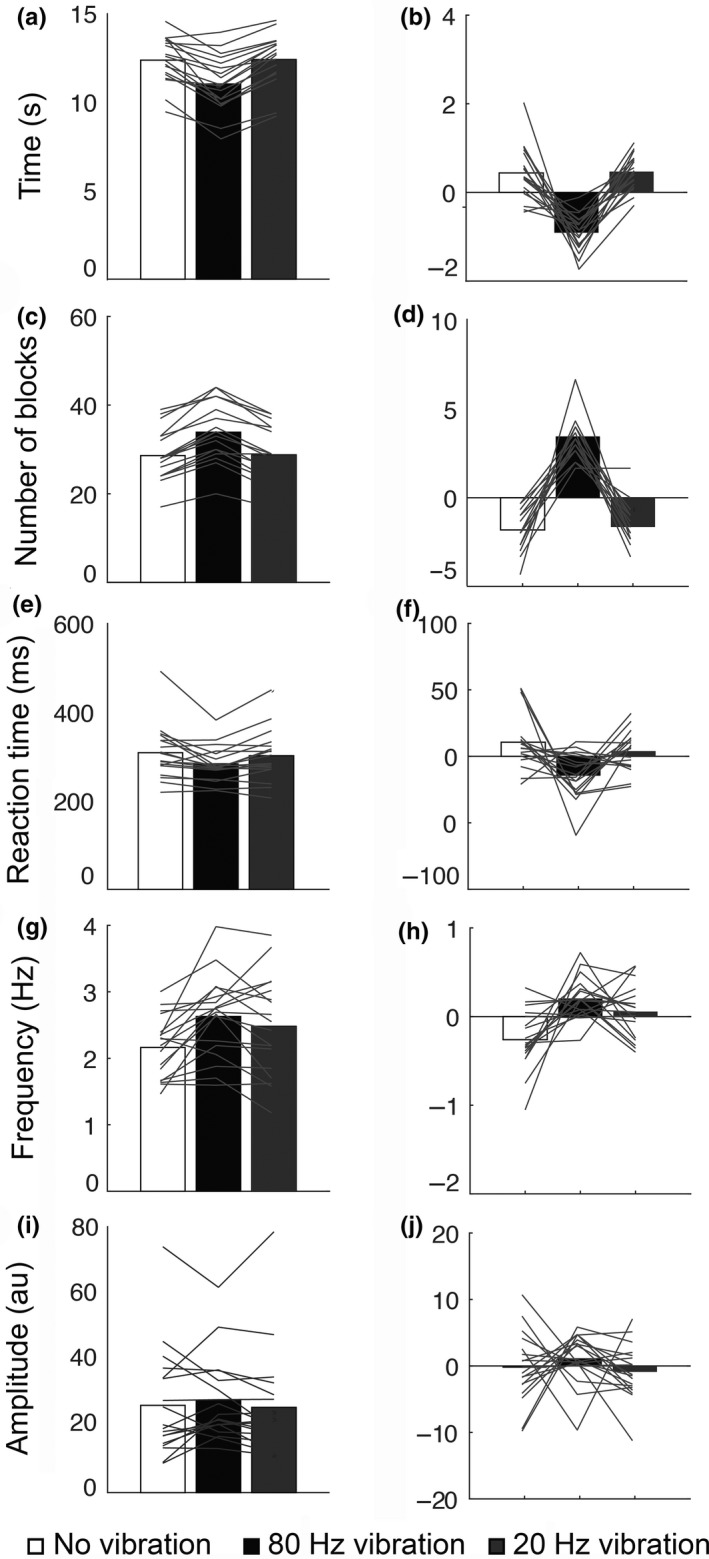
Results of Experiment 3. Healthy subjects show improved motor performance following 80 Hz vibration but not to 20 Hz vibration. Bar graphs show the mean and the corrected mean of completion time for the nine‐hole peg task (a, b), of the number of the blocks moved in 30 s on the box and blocks task (c, d), of the reaction time task (e, f), of tapping frequency measured with a cyberglove (g, h) and of tapping amplitude (i, j). 80 Hz vibration (black bars). 20 Hz vibration (grey bars). No vibration (white bars)

Repeated measures ANOVA revealed a significant main effect of condition on the performance of the nine‐hole peg test, (*F*_2,34_ = 32.025, *p* = 0.000, *η* ^2^ = 0.653). Post hoc pairwise comparisons, corrected for multiple comparisons, revealed a significant difference between mean movement speed following 80 Hz vibration (11.01 ± 1.58 s) and no vibration (12.35 ± 1.31 s), (*t*_17_ = 5.899, *p* = 0.000), as well as between 80 Hz vibration and 20 Hz vibration (12.38 ± 1.46 s), (*t*_17_ = −11.064, *p* = 0.000). There was no significant difference in mean movement speed following 20 Hz vibration and no vibration, (*t*_17_ = −1.139, *p* = 0.8; Figure 4a,b).

For the box and blocks test, a repeated measures ANOVA showed a significant main effect of condition on motor performance, (*F*_2,34_ = 74.478, *p* = 0.000, *η* ^2^ = 0.814). Post hoc pairwise comparisons, corrected for multiple comparisons, revealed a significant difference between the number of cubes moved following 80 Hz vibration (34 ± 7 boxes) and no vibration (29 ± 5 boxes), (*t*_17_ = −11.228, *p* = 0.000), as well as between 80 Hz vibration and 20 Hz vibration (29 ± 6 boxes), (*t*_17_ = 10.409, *p* = 0.000). There was no significant difference between the performance of the test following baseline and 20 Hz vibration, (*t*_17_ = −0.325, *p* = 0.7; Figure 4c,d).

For the reaction time test, a repeated measures ANOVA showed a significant main effect of condition on motor performance, (*F*_2,34_ = 6.416, *p* = 0.004, *η* ^2^ = 0.274). Post hoc pairwise comparisons, corrected for multiple comparisons, revealed a significant difference in reaction time following 80 Hz vibration on the right wrist (283.17 ± 39.408 ms) and no vibration (308.83 ± 60.601 ms), (*t*_17_ = 3.044, *p* = 0.007), as well as between 80 Hz vibration and 20 Hz vibration (301.67 ± 57.455 ms), (*t*_17_ = −3.128, *p* = 0.006). There was no significant difference between the performance of the test following baseline and 20 Hz vibration, (*t*_17_ = 0.894, *p* = 0.3; Figure 4e,f).

In this group, the tapping test was performed and the kinematics of the hand movements were measured using a cyber glove. Two measures were calculated: the amplitude and the frequency of the taps. A repeated measures ANOVA revealed no significant main effect of condition on the amplitude of the tapping, (*F*_2,34_ = 0.663, *p* = 0.5, *η* ^2^ = 0.038; Figure 4i,j). However, a repeated measures ANOVA showed a significant main effect of condition on the frequency of the tapping, (*F*_2,34_ = 7.838, *p* = 0.002, *η* ^2^ = 0.316; Figure 4g,h). Post hoc pairwise comparisons, corrected for multiple comparisons, revealed a significant difference between the tapping frequency following 80 Hz vibration (2.63 Hz ± 0.61) and no vibration (2.16 Hz ± 0.46), (*t*_17_ = −3.981, *p* = 0.001), as well as between 80 Hz vibration and 20 Hz vibration (2.48 Hz ± 0.74), (*t*_17_ = 2.278, *p* = 0.001). There was no significant difference between the performance of the test following 20 Hz vibration and no vibration, (*t* = −1.464, *p* = 0.1).

### 3.4 Experiment 4

The previous three experiments demonstrated significant modulations in different movement parameters of the right hand following 30 s of 80 Hz vibration applied to the right wrist. This is consistent with the hypothesis tested that increasing noise in the somatosensory afferent signal would result in faster movements and movement initiation in young healthy controls. In Experiment 4, we tested the hypothesis that vibration at 80 Hz applied to the right wrist would improve motor performance in participants with PD.

**Figure 5:**
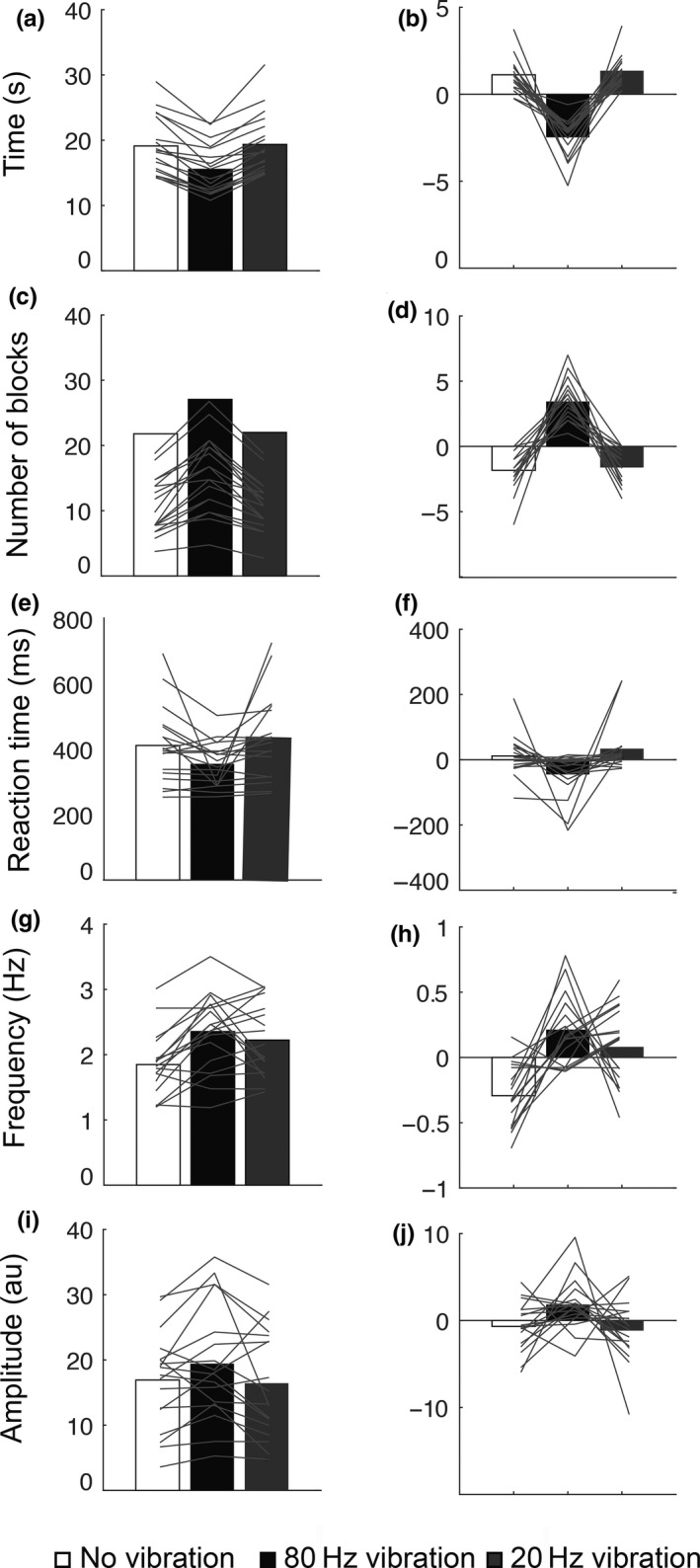
Results of Experiment 4. Parkinson's disease patients show improved motor performance following 80 Hz vibration but not 20 Hz. Bar graphs show the mean and the corrected mean of completion time for the nine peg‐hole (a, b), of the number of the blocks moved in 30 s (c, d), of the reaction time task (e, f), of tapping frequency (g, h) and tapping amplitude (i, j)

A repeated measures ANOVA revealed a significant main effect of condition on the performance of the nine‐hole peg test, (*F*_2,34_ = 58.355, *p* = 0.000, *η* ^2^ = 0.774). Post hoc pairwise comparisons revealed a significant difference between mean movement speed following 80 Hz vibration (15.52 ± 3.82 s) and no vibration (19.12 ± 4.45 s), (*t*_17_ = 8.229, *p* = 0.000) as well as 80 Hz vibration and 20 Hz vibration (19.35 ± 4.65 s), (*t*_17_ = −9.485, *p* = 0.000). There was no significant difference between mean movement speed following no vibration and 20 Hz vibration, (*t*_17_ = −0.682, *p* = 0.5; Figure 5a,b).

Furthermore, for the blocks and box test, a repeated measures ANOVA showed a significant main effect of condition on motor performance, (*F*_2,34_ = 45.234, *p* = 0.000, *η* ^2^ = 0.727). Post hoc pairwise comparisons revealed a significant difference between the number of cubes moved following 80 Hz vibration (28 ± 5 boxes) and no vibration (22 ± 4 boxes), (*t*_17_ = −7.262, *p* = 0.000), as well as between 80 Hz vibration and 20 Hz vibration (22 ± 4 boxes), (*t*_17_ = 8.321, *p* = 0.000). There was no significant difference between the performance of the test following no vibration and 20 Hz vibration, (*t*_17_ = −0.416, *p* = 0.6; Figure 5c,d).

Regarding reaction time task, a repeated measures ANOVA showed a significant main effect of condition on the reaction time, (*F*_2,34_ = 4.078, *p* = 0.02, *η* ^2^ = 0.193). Post hoc pairwise comparisons revealed no significant difference between mean reaction time following 80 Hz vibration (355.89 ± 67.77 ms) and no vibration (412.28 ± 116.53 ms), (*t*_17_ = 2.310, *p* = 0.03) and a significant difference between 80 Hz vibration and 20 Hz vibration (434.61 ± 129.81 ms), (*t*_17_ = −2.496, *p* = 0.002). There was no significant difference between the performance of the test following 20 Hz vibration and no vibration, (*t*_17_ = −0.775, *p* = 0.4; Figure 5e,f).

A repeated measures ANOVA showed a significant main effect of condition on the frequency of tapping, (*F*_2,34_ = 11.623, *p* = 0.000, *η* ^2^ = 0.406). Post hoc pairwise comparisons corrected for multiple comparisons revealed a significant difference between tapping frequency following 80 Hz vibration (2.354 Hz ± 0.58) and no vibration (1.848 Hz ± 0.48), (*t*_17_ = −5.313, *p* = 0.000), but not between 80 Hz vibration and 20 Hz vibration (2.223 Hz ± 0.56), (*t*_17_ = 1.090, *p* = 0.2). There was a significant difference between the frequency of tapping following 20 Hz vibration and no vibration (*t*_17_ = −3.428, *p* = 0.003; Figure 4g,h). There was a statistical trend of the effect of condition on the amplitude of the tapping, (*F*_2,34_ = 3.090, *p* = 0.05, *η* ^2^ = 0.154; Figure 4i,j). There was a significant difference between the amplitude of the tapping following 80 Hz vibration (19.42 a.u. ± 8.85) and no vibration (16.91 a.u. ± 7.35; *t*_17_ = −2.377, *p* = 0.02). There was a statistical trend in the difference between the amplitude of the tapping following 80 Hz vibration and 20 Hz (16.43 a.u. ± 8.27; *t*_17_ = 2.077, *p* = 0.05). There was no difference between no vibration condition and 20 Hz vibration (*t*_17_ = 0.356, *p* = 0.7).

In order to determine if there were any significant differences in motor performance following 80 Hz vibration between the 18 healthy subjects that participated in Experiment 3 and the 18 PD patients, a repeated measures ANOVA was conducted for each behavioural task with group as a between subject's factor and condition (no vibration, 80 Hz vibration to the right wrist and 20 Hz vibration) as a within‐subjects factor. The motor performance of healthy controls was significantly different from PD patients on the nine‐hole peg task (*F*_1,34_ = 33.906, *p* = 0.000, *η* ^2^ = 0.499), the box and blocks test (*F*_1,34_ = 14.637, *p* = 0.001, *η* ^2^ = 0.301), the simple reaction time task (*F*_1,34_ = 20.481, *p* = 0.000, *η* ^2^ = 0.376) and amplitude of tapping (*F*_1,34_ = 5.471, *p* = 0.02, *η* ^2^ = 0.139), but not in the frequency of tapping (*F*_1,34_ = 2.774, *p* = 0.1, *η* ^2^ = 0.075).

Overall, PD patients produced slower movements than healthy controls (Table 2).

**Table 2:** Motor performance of the PD patients and the healthy subjects recruited in Experiment 3

	PD patients	Healthy subjects
Peg‐hole task^a^		
No vibration	19.12 ± 4.45	12.35 ± 1.31
80 Hz vibration	15.52 ± 3.82	11.01 ± 1.58
20 Hz vibration	19.35 ± 4.65	12.38 ± 1.46
Box and blocks test^b^		
No vibration	22 ± 4	29 ± 5
80 Hz vibration	28 ± 5	34 ± 7
20 Hz vibration	22 ± 4	29 ± 6
Reaction time^c^		
No vibration	412.28 ± 116.53	308.33 ± 60.61
80 Hz vibration	355.89 ± 67.77	283.16 ± 39.41
20 Hz vibration	434.61 ± 129.81	301.66 ± 57.45
Tapping frequency^d^		
No vibration	1.848 ± 0.48	2.16 ± 0.46
80 Hz vibration	2.354 ± 0.58	2.63 ± 0.61
20 Hz vibration	2.223 ± 0.56	2.48 ± 0.74
Tapping amplitude^e^		
No vibration	16.91 ± 7.35	26.27 ± 16.23
80 Hz vibration	19.43 ± 8.85	27.68 ± 12.46
20 Hz vibration	16.43 ± 8.27	25.65 ± 16.10

Notes

a Completion time in seconds.

b Number of boxes moved in 30 s.

c Completion time in milliseconds.

d Frequency in Hz.

e Amplitude in a.u.

In support of previous results, there was a significant main effect of condition in all motor tasks (all *p* < 0.003) except the amplitude of tapping (*p* = 0.06). The interaction between condition and group was not significant for any of the motor tasks (*p* > 0.05) suggesting the magnitude of improvement following 80 Hz vibration was similar between the groups.

### 3.5 Experiment 5

The previous four experiments have demonstrated that right‐handed movements made immediately after 30 s of 80 Hz vibration applied to the right wrist are performed significantly faster than after no vibration or after vibration at a lower frequency, 20 Hz. Here we tested the hypothesis that 80 Hz vibration would result in a decrease in beta oscillatory power over sensorimotor cortex contralateral to the wrist where the vibration was applied.

**Figure 6:**
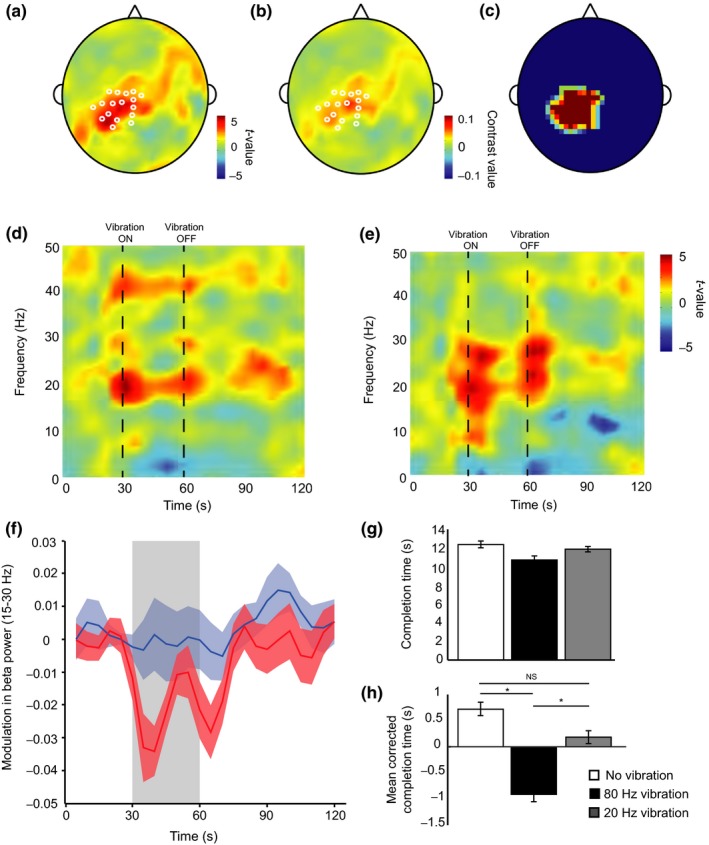
Results of Experiment 5. Beta power over sensorimotor cortex decreased at the onset and offset of 80 Hz peripheral vibration. (a, b) Topography of the EEG activity averaged over 15–30 Hz during vibration. White circles show electrodes which showed a significant attenuation of beta power during vibration superimposed on (a) the *t* statistic scalp image and (b) the contrast image. (c) ROI selected for subsequent time‐frequency analyses. (d, e) Time‐frequency *t* statistic images within an ROI over contralateral sensorimotor cortex for (d) the difference between 80 and 20 Hz vibration conditions, and (e) the difference between 80 Hz vibration and a baseline window. Data are shown in the period prior to, during and following vibration. (f). Time course of beta power modulation for 80 Hz vibration (red) and 20 Hz vibration (blue). The shaded block represents the time when vibration was on. (i, j) Bar graphs showing (g) the completion time (h) and mean‐corrected completion time for the nine‐hole peg task carried out by these subjects. [Colour figure can be viewed at http://wileyonlinelibrary.com]

An initial analysis tested the hypothesis that there was a significant attenuation of power in the 15–30 Hz range during the period when 80 Hz vibration was applied compared with 20 Hz vibration. Beta power was significantly lower at the onset of 80 Hz vibration compared with onset of 20 Hz vibration specifically at electrodes overlying the left sensorimotor cortex (peak voxel *t*_1,16_ = 4.87, *p* < 0.05 corrected for FWE; Figure 6a–c).

Subsequent analyses focused on the average time‐frequency plots overlying the electrodes of interest where beta power was significantly attenuated. The time course of beta power modulation averaged over the electrodes of interest and across the beta frequency range (15–30 Hz) revealed that beta power was attenuated at the onset and offset of the 30 s vibration period (Figure 6f). To investigate this further, the time‐frequency plots averaged over the electrodes of interest were compared. This analysis revealed a significant attenuation of oscillatory power at 20 Hz at the onset of 80 Hz vibration compared with 20 Hz vibration (*t*_1,16_ = 5.13, *p* < 0.05 corrected for FWE; Figure 6d). One possible explanation for these results is that the decrease in power in the beta frequency range reflects an increase in beta power during the 20 Hz vibration, reflecting the power at the frequency of vibration. To exclude this hypothesis, we tested whether there was a significant attenuation of power in the 80 Hz condition compared to baseline. This analysis revealed two clusters of significant attenuation one at the onset of the vibration (*t*_1,16_ = 4.7 peak at 27 Hz and one at the offset (*t*_1,16_ = 4.59 peak at 23 Hz; Figure 6e).

The 18 subjects in Experiment 5 also completed the nine‐hole peg test. A repeated measures ANOVA revealed a significant main effect of condition on the right wrist on the performance of the nine‐hole peg test, (*F*_2,34_ = 32.758, *p* = 0.000, *η* ^2^ = 0.658). Post hoc pairwise comparisons corrected for multiple comparisons revealed a significant difference between mean movement speed following 80 Hz vibration (10.71 ± 1.40 s) and no vibration (12.36 ± 1.04 s), (*t*_17_ = 7.480, *p* = 0.000) as well as 80 Hz vibration and 20 Hz vibration (11.87 ± 1.22 s), (*t*_17_ = −5.529, *p* = 0.000). There was no significant difference between mean movement speed following no vibration and 20 Hz vibration, (*t*_17_ = 2.502, *p* = 0.7; Figure 6g,h). In addition, to better highlight the reproducibilty of effects of vibration on the different motor tasks we analysed the data across the different tasks with experiment as a factor as well as analysing the effect of vibration within each experiment. (see supplementary Figure S1 and Table S1).

## 4 DISCUSSION

The current study aimed to provide evidence consistent with the hypothesis that increasing proprioceptive uncertainty would lead to improvements on a number of motor control tasks. Here, a peripheral vibrating stimulus at 80 Hz was used to change the proprioceptive signal and in doing so change alter the uncertainty of the afferent signal. As hypothesised, 30 s of 80 Hz peripheral vibration applied to the right wrist of a total of 54 healthy controls reproducibly improved performance related to movement speed across four separate experiments on a number of motor control tasks. Improved performance on all motor tasks (except the amplitude of finger tapping) was also seen for a sample of 18 PD patients ON medication. Data investigating the effect of vibration on oscillatory activity revealed a significant decrease in beta oscillatory activity (15–30 Hz) over the contralateral sensorimotor cortex at the onset and offset of 80 Hz vibration. In contrast, peripheral vibration at 20 Hz had no effect on motor performance and caused no modulation in beta oscillatory activity. Overall, it is clear that peripheral vibration at 80 Hz improved motor performance on a variety of motor control tasks and this improvement may have been driven by a modulation of beta oscillatory activity over sensorimotor cortex. In the current study, there was only a significant effect of vibration on behavioural performance at 80 Hz.

According to active inference, in order to initiate a movement, we must decrease the certainty in our current sensory state through attenuation of the afferent signal (Brown, Adams, Parees, Edwards, & Friston, 2013; Friston et al., 2011). It is hypothesised that reducing the synaptic gain on superficial pyramidal cells, thought to transmit bottom‐up prediction errors causes this attenuation and thus provides the necessary gateway to allow movement initiation to occur (Bastos et al., 2012; Friston, Bastos, Pinotsis, & Litvak, 2015). Here we sought to artificially modulate the certainty of the proprioceptive afferent signal using high‐frequency peripheral vibration. Previous research has shown that peripheral vibration at 80 Hz impairs performance on a number of proprioceptive tasks (Cordo et al., 1995, 2005; Inglis & Frank, 1990; Tsay et al., 2016), which is thought to be driven by increasing uncertainty in the proprioceptive input. Indeed, high‐frequency vibration produces the illusion that the relevant muscle is contracting in the absence of any EMG activity by transmitting incorrect kinesthetic information to the brain and spinal cord such that the brain is uncertain about the relative position of the limb (Goodwin et al., 1972; McCloskey, 1973). Moreover, previous studies have demonstrated that high‐frequency peripheral vibration leads to sensory attenuation, as indicated by a decrease in the amplitude of SEPs evoked by electrical stimulation of the afferent nerve. Peripheral vibration at 60 Hz causes an attenuation of early components of the cortical and cervical SEP (Abbruzzese et al., 1980; Cohen & Starr, 1985); yet, 50 Hz cutaneous vibration between the thumb and finger and 20 Hz vibration at the wrist does not produce significant sensory attenuation (Kakigi & Jones, 1986; Legon & Staines, 2006). Here we have demonstrated that high‐frequency peripheral vibration at 80 Hz, and not 20 Hz, decreases reaction time and completion time on a number of behavioural tasks. Based on previous empirical and theoretical work, we hypothesise that this is due to an increase in proprioceptive uncertainty causing an attenuation of the afferent input. However, future work will be required to fully determine and characterise the causal relationship between peripheral vibration and estimates of somatosensory uncertainty.

Interestingly in the current study, there was only a significant effect of vibration on behavioural performance at 80 Hz and not 20 Hz. Previous literature exploring the neurophysiological effect of peripheral vibration suggests that this is likely due to the mechanical stimulation of muscle spindles, most sensitive to high‐frequency stimulation around 80–120 Hz, which in turn readily activate 1a motor afferents (Roll et al., 1989). These afferent fibres provide an essential source of information about the dynamic position of the muscle necessary for optimal proprioceptive feedback. Neuroimaging studies have shown that high‐frequency vibration activates areas involved in sensory integration of information necessary for movement planning (Casini et al., 2006; Naito, Ehrsson, Geyer, Zilles, & Roland, 1999; Romaiguère, Anton, Roth, Casini, & Roll, 2003; Smith & Brouwer, 2005). It has been suggested that vibration at high frequencies improves motor performance by increasing top‐down proprioceptive feedback control, by attenuating bottom‐up sensory input (in line with active inference), and increasing the excitability of the sensorimotor cortex (Conrad, Scheidt, & Schmit, 2011). Indeed, the novel finding in the current study that beta oscillatory activity over sensorimotor cortex decreased in response to 80 Hz vibration supports this. Furthermore, as previously described high‐frequency vibration modulates SEP attenuation; however, ischaemic block of 1a motor afferents has been shown to eradicate SEP attenuation (Abbruzzese et al., 1980). This supports the hypothesis that activating 1a motor afferents specifically may be necessary for sensory attenuation. Although we did not directly record from 1a afferents, we hypothesise that the improvements in behaviour seen specifically following 80 Hz vibration were due to an increased firing of 1a afferents, which modulated beta oscillatory activity over sensorimotor cortex and placed the sensorimotor system in a “ready‐to‐move” state. However, we are aware that there are other fibre endings within the muscle that would have been simultaneously activated, therefore the contribution of other afferent inputs cannot be ruled out. Combined microneurography and EEG studies are required to confirm this relationship between 1a afferent firing rate and sensorimotor beta oscillatory activity to support this hypothesis.

It is well established that beta oscillations over sensorimotor cortex decrease prior to and during movement (Gastaut, 1952; Jasper & Penfield, 1949) and this modulation broadly correlates with the time course of SEP attenuation (Starr & Cohen, 1985). Previous research has also proposed that uncertainty in motor control is correlated with modulations in beta oscillatory power recorded from EEG overlying the sensorimotor cortex, with increasing uncertainty correlated with decreased beta oscillatory power (Palmer et al., 2016; Tan, Wade, & Brown, 2016). This implicates beta oscillatory activity as a promising candidate for this gating mechanism. Indeed, in the current study, there was a decrease in beta oscillatory activity at the onset and offset of 80 Hz peripheral vibration, but not 20 Hz. Here we have shown in healthy subjects that increasing proprioceptive uncertainty through peripheral vibration causes an attenuation in beta power in the motor system, suggesting a link between beta power and the estimate of somatosensory certainty. Patients with Parkinson's disease have abnormally high beta oscillatory activity within the motor system, which correlates with symptom severity (Brown, 2007; Brown et al., 2001; Kühn, Kupsch, Schneider, & Brown, 2006). It has previously been proposed that the bradykinetic symptoms of PD are linked to a failure to attenuate their somatosensory precision, and in turn reduce beta power, in order to allow movement to occur (Friston et al., 2011). The results here extend this and point to a possible link between the estimate of somatosensory certainty and beta power in healthy controls and in PD patients.

The work here highlights the potential use of high‐frequency vibration as a noninvasive treatment for PD patients as an adjunct to dopaminergic medication. However, more work is needed to identify the specificity of this effect to 80 Hz stimulation frequency, to explore how long improvements in motor control last and to specifically investigate a clinically significant effect in this patient group before any claims of treatment efficacy can be made. The use of peripheral vibration to treat symptoms of movement disorders is not a novel concept and was first realised with Charcot's “Vibrating Chair” in 1982 (Charcot, 1982). Following this there have been a number of studies investigating the clinical efficacy of peripheral vibration, particularly of the whole body (Arias, Chouza, Vivas, & Cudeiro, 2009; Chouza, Arias, Viñas, & Cudeiro, 2011; Ebersbach, Edler, Kaufhold, & Wissel, 2008; Haas, Turbanski, Kessler, & Schmidtbleicher, 2006; Kapur, Stebbins, & Goetz, 2012; King, Almeida, & Ahonen, 2009); however, the results have been inconsistent due to differences in the vibration protocol used, the muscles targeted, the behaviours being measured and the patient groups studied. In particular, there have been limited studies that have shown an improvement in behavioural performance in healthy controls following vibration, which is likely due to healthy controls performing at ceiling on the behavioural tasks used.

In conclusion, the current study demonstrated that high‐frequency vibration applied to the periphery improved motor control on a number of motor tasks in healthy subjects and patients with PD. This was associated with a decrease in beta oscillatory activity over sensorimotor cortex at the onset and offset of vibration. These results are consistent with a novel theoretical account of motor initiation, namely that modulating uncertainty of the proprioceptive afferent signal improves motor performance potentially by gating the incoming sensory signal and allowing for top‐down proprioceptive predictions that incite movement to be more readily fulfilled. We further hypothesise that this gating is mediated by beta oscillatory activity over sensorimotor cortex.

## CONFLICT OF INTEREST

The authors declare no competing financial interests.

## AUTHOR CONTRIBUTIONS

AM, CP, JK designed the experiment; AM, CP, JK performed data acquisition; AM, CP, JK analysed data; AM, CPA. TF, PK, PL, MEJ and JK wrote the manuscript.

## DATA ACCESSIBILITY

The data are available from the authors. They are not uploaded into public repositories, because the ethics consent did not include this.
