# Supplementary material for: High‐frequency peripheral vibration decreases completion time on a number of motor tasks
Source: Eur J Neurosci. 2018 Aug 6;48(2):1789–802. doi: 10.1111/ejn.14050 (PMC6175240; doi:10.1111/ejn.14050)

Supplement Materials

Fig. 1 A: Omnibus statistics for 9 Peg Hole Test

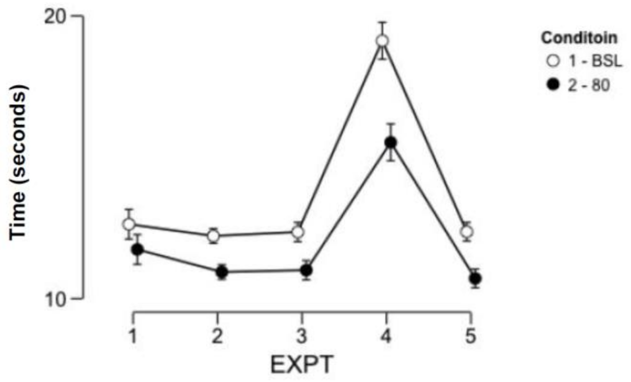

Fig. 1 B: Omnibus statistics for box and blocks task

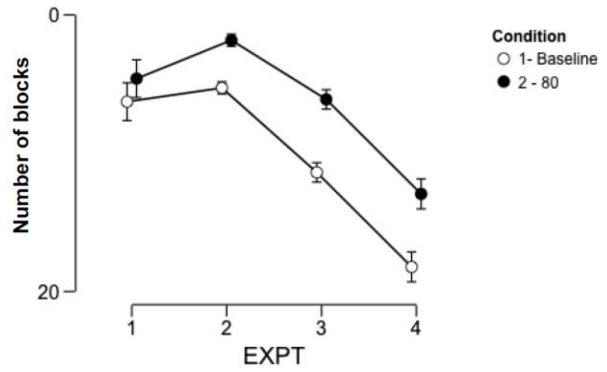

Fig. 1 C: Omnibus statistics for Reaction Time Task

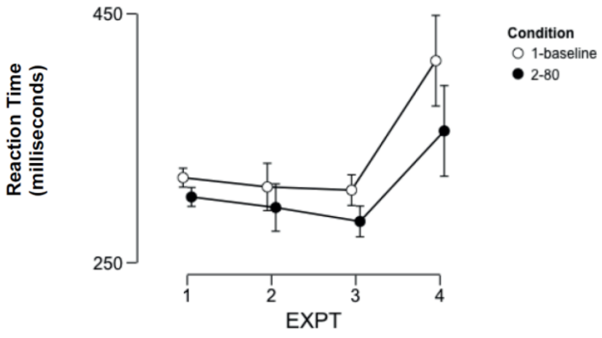

Supplement: Supplementary file 1 [file EJN-48-1789-s001.pdf]
