# Supplementary material for: High‐frequency peripheral vibration decreases completion time on a number of motor tasks
Source: Eur J Neurosci. 2018 Aug 6;48(2):1789–802. doi: 10.1111/ejn.14050 (PMC6175240; doi:10.1111/ejn.14050)
Supplement: Supplementary file 2 [file EJN-48-1789-s002.docx]

|  | **9 Peg- hole test** | | **Box and blocks test** | | **Reaction time test** | | **Frequency of**  **fingers tapping** | | **Amplitude of fingers tapping** | |
| --- | --- | --- | --- | --- | --- | --- | --- | --- | --- | --- |
|  | ANOVA | t-test baseline vs 80 Hz | ANOVA | t-test baseline vs 80 Hz | ANOVA | t-test baseline vs 80 Hz | ANOVA | t-test  baseline vs  80 Hz | ANOVA | t-test  baseline vs  80 Hz |
| **Experiment 1** |  | t (17) =2.532  p = 0.02 |  | t (17)=1.822  p = 0.08 |  | t (17)=3.046  p = 0.007 |  |  |  |  |
| **Experiment 2** | F(2,34)=31.686  p = 0.000 | t (17) =7.351  p = 0.000 | F(2,34)=116.978  p = 0.000 | t(17)=11.717  p = 0.000 | F(2,34)=1.856  p = 0.1 | t (17) =1.3  p = 0.2 |  |  |  |  |
| **Experiment 3** | F(2,34)=32.025  p = 0.000 | t (17) =5.899 p = 0.000 | F (2, 34) = 74.478  p = 0.000 | t(17)= - 11.228  p = 0.000 | F(2, 34) = 6.416  p = 0.004 | t (17) =3.044 p = 0.007 | F(2,34) =7.838  p = 0.002 | t (17)= -3.981  p = 0.001 | F(2,34)= 0.663  p = 0.5 | t(17) = - 0.735  p = 0.4 |
| **Experiment 4** | F (2, 34) =58.355  p = 0.000 | t (17) = 8.229  p = 0.000 | F (2, 34) = 45.234  p = 0.000 | t (17) = -7.262  p = 0.000 | F (2, 34) = 4.078  p = 0.02 | t (17) = 2.310  p = 0.03 | F (2, 34) = 11.623 p = 0.000 | t (17) = -5.313  p = 0.000 | F (2, 34) = 3.090  p = 0.05 | t (17) = -2.377  p = 0.02 |
| **Experiment 5** | F (2,34) = 32.758 p = 0.000 | t (17) = 7.480  p = 0.000 |  |  |  |  |  |  |  |  |
| **Omnibus** | F (1, 85) = 172.06  p<0.001 |  | F (1,68) = 146,787 p<0.001 |  | F (1, 68) = 15.146  p<0.001 |  |  |  |  |  |
