# Supplementary material for: High‐frequency peripheral vibration decreases completion time on a number of motor tasks
Source: Eur J Neurosci. 2018 Aug 6;48(2):1789–802. doi: 10.1111/ejn.14050 (PMC6175240; doi:10.1111/ejn.14050)
Supplement: Supplementary file 3 [file EJN-48-1789-s003.docx]

High frequency peripheral vibration decreases completion time on a number of motor tasks

Macerollo, Antonella; Palmer, Clare; Foltynie, Thomas; Korlipara, Prasad; Limousin, Patricia; Edwards, MJ; Kilner, James

_____________________________________________________________________________________

| Review timeline: | Submission date: 24/08/17  Editorial Decision: Major Revision  Revision Received: 30/12/17  Editorial Decision: Rejection  Revision Received: 30/15/18  Accepted: 18/06/18 |  |
| --- | --- | --- |
|  |  |  |

Editor: Ali Mazaheri

Reviewer 1: Marco Bove

Reviewer 2: Davide Martino

_____________________________________________________________________________________

1st Editorial Decision	22/10/2017

_____________________________________________________________________________________

High frequency peripheral vibration decreases completion time on a number of motor tasks

Dear Dr. Macerollo,

We have now received the critiques of our two expert external reviewers and your paper has also been thoroughly assessed by the editors-in-chief. As you will read, we have divided opinions regarding the merits of the work, and both reviews have provided a set of clearly articulated criticisms that will require your attention before we can consider proceeding with this paper. In general, the revisions required are of the major variety and you will need to be fully responsive to them. For example, you will need to do a much better job of describing the experimental paradigm and a considerably more detailed treatment of what was asked of your participants. This is also the case with regard to your analytic approach. You need to describe clearly and in detail, as well as justifying, the choices you made with regard to the analyses conducted. Your illustrations also need better explanation and more detail (note that their numbering is off in places).  It was also felt that a more comprehensive discussion of all of the results would be required. Your paper also needs to be thoroughly checked for typos, punctuation errors and general grammar – the attentions of a professional copy-editor would be advisable.

Please also attend to the following issues in your revision:
1) Provide the full name of ethics committee
2) Please avoid reporting your statistical p-values as inequalities – write them out fully where possible.
3) We require specific statements in the main text about a) Author contributions, 2) Conflict of Interest, and c) a data sharing statement
4) Your references have what we refer to as the 'endnote problem' – please correct all formatting issues and check them carefully
5) Replace your bar charts with scatterplots or some other more informative hybrid depictions - see http://onlinelibrary.wiley.com/doi/10.1111/ejn.13400/epdf
6) Your figures need to be clearly labelled so readers know which is which

When revising the manuscript, please embolden or underline major changes to the text so they are easily identifiable and DO NOT leave 'track change' formatting marks in your paper.  Please ensure that you provide a text and a figure file for the Graphical Abstract (as detailed in the instructions below).  When carrying out your revisions please refer to the checklist below and visit the EJN author guidelines at www.ejneuroscience.org

When finalized, please upload your complete revised manuscript onto the website, as a Word file (.doc, or .docx).  Please also ensure that a complete set of tables and figures is included as separate files, even if these have not changed from the originals.  At this stage it is necessary to provide high resolution figures.  Please see important instructions below.

Please go into https://mc.manuscriptcentral.com/ejn - Author Centre - manuscripts with decisions where you will find a 'create a revision' link under 'actions'. We ask that you please indicate the way in which you have responded to the points raised by the Editors and Reviewers in a letter. Please upload this response letter as a separate Word (.doc or PDF) file using the file designation "Authors' Response to Reviewers" when uploading your manuscript files.  Please DO NOT submit your revised manuscript as a new one.  Also, please note that only the  Author who submitted the original version of the manuscript should submit a revised version.

If you are able to respond fully to the points raised, we would be pleased to receive a revision of your paper within 12 weeks.

Thank you for submitting your work to EJN.

Kind regards,

John Foxe & Paul Bolam
co-Editors in Chief, EJN


Reviews:

Reviewer: 1

Comments to the Author
This is an interesting study exploring the effect of high frequency vibratory stimulation upon movement initiation and speed and an EEG correlate of this, beta power attenuation, in healthy and Parkinson’s disease subjects. The underlying mechanisms hypothesized for this effect is the increase in uncertainty of proprioceptive afferent input immediately after motor initiation. The results confirm the a priori hypotheses.
I have a few comments to improve the manuscript. The authors should carefully edit the manuscript to correct punctuation errors and a few typos.

INTRODUCTION
The kinesthetic illusion generated by high frequency vibratory stimulation consists of a false sensation of muscle contraction, not lengthening. This is correctly reformulated in the first paragraph of the Discussion.

METHODS
Experiments 1 and 2
Why was Experiment 1 reported separately, if the only difference from the first is the addition of a 3rd condition of vibratory stimulation over the left wrist, and results of Experiment 2 incorporate results of Experiment 1? Moreover, the results of the Box and blocks test are different between Experiment 1 and Experiment 2 with respect to the comparison between the “absent vibratory stimulation” and the “80 Hz vibration”, which is difficult to interpret (although the direction of the effect is the same).
On page 5, line 2: please define the interstimulus interval (which are the stimuli separated by this interval? The reaction time task has 1 stimulus per trial, but the indicated ISIs are very small to mean time elapsing between trials).
The last sentence refers to the 20 Hz vibration condition, which was not part of this Experiment. This seems an editing mistake?!

Experiment 3
Explain here the rationale of the 20 Hz vibratory stimulation condition.

DISCUSSION
Can the authors speculate on why beta attenuation occurred both at the onset and at the offset of the 80 Hz vibratory stimulation? What happened throughout the stimulation: did the beta power return to baseline, and was there any effect of condition?

Can the authors comment on whether they expect that the effect of high frequency vibratory stimulation in increasing uncertainty of proprioceptive afferent input has a topographical specificity? Also, do the authors expect a difference of effect upon voluntary, non-automatic movements as opposed to automatic motor programmes, e.g. gait? Reproducing the same effect in the lower limbs would be of enormous interest and could support this as an adjunctive intervention for freezing of gait, which is a failure of gait initiation.


Reviewer: 2

Comments to the Author
I think that this work needs some massive revision and that is not ready for publication in the present version. In my opinion, the experiments and the results do not support the hypothesis of the authors. Following some points that deserve authors’ attention.

-Authors should provide more information and details about the experimental phases, because some of them are not clear. For example, could the timeframes of the tasks be described? Did motor tasks begin as soon as the vibration stopped? This is not clearly explained. If so, how does the reaction time task worked? Did subjects repeat this task 3 times in total or per jitter duration? Did you find any differences between these conditions? What kind of task did subjects do with the glove? Did they only had to tap thumb and index finger? What were the instructions?
Did subjects perceived kinesthetic illusion or not?

-I think that the healthy young group of subjects of Experiment 3 wasn’t the right control for the older group of PD patients.  

-Author should describe in detail what kind of analysis they performed for each experiment in the Data Analysis paragraph underlining the factors they use in every analysis. They also had to explain how the corrected means were calculated, if they chose to use them.

-How do authors explain the fact that some performances significantly improved in some experiment and does not in others? I’m referring to, as an example, Reaction time task results in exp1 and 2. I think they must comment on this fact.

-I think that figure 4 refers to experiment 4, and not experiment 3 where it is cited.

-Figures lacks details like titles and axis, but “raw” figures and corrected mean figures are redundant. Why did authors indicate statistical significance over the means graphs if they performed the statistical analysis over raw values, indicated in the main text?

I think that the discussion needs some theoretical revision, but in general authors have to explain the findings of all their experiment more in detail and comment the literature they cite carefully.

_____________________________________________________________________________________

Authors’ Response	30/12/2017

_____________________________________________________________________________________

Editors’ comments

1) You will need to do a much better job of describing the experimental paradigm and a considerably more detailed treatment of

what was asked of your participants. This is also the case regarding your analytic approach. You need to describe clearly

and in detail, as well as justifying, the choices you made regarding the analyses conducted. Your illustrations also need better

explanation and more detail (note that their numbering is off in places). It was also felt that a more comprehensive discussion of

all the results would be required. Your paper also needs to be thoroughly checked for typos, punctuation errors and general

grammar – the attentions of a professional copy-editor would be advisable.

Page 1 of 52European Journal of Neuroscience

For Peer Review

RE: We thank the editor for his useful comments. We have reviewed the manuscript and we have added details regarding the protocols

and analysis. We have reviewed our illustrations and we added some details. The discussion has been completed with a more

comprehensive view on our results. The typos, punctuation errors and general grammar have been reviewed. The English Authors

reviewed the language errors.

2) Provide the full name of ethics committee

RE: The ethics committee involved in the study was the East of Scotland Research Ethics Service (EoSRES). We have added the full name

in the manuscript (page 5, line 126-127).

3) Please avoid reporting your statistical p-values as inequalities – write them out fully where possible.

RE: We thank the editor for pointing out this limitation of our manuscript. We have now reported the statistical p-values as full number

Page 2 of 52 European Journal of Neuroscience

For Peer Review

across the results section.

4) We require specific statements in the main text about a) Author contributions, 2) Conflict of Interest, and c) a data sharing

statement

RE: We have now added the specific statements in the main text.

5) Your references have what we refer to as the 'endnote problem' – please correct all formatting issues and check them carefully

RE: We thank the Editor for pointing out this issue. We have reviewed the references list.

6) Replace your bar charts with scatterplots or some other more informative hybrid depictions – see

http://onlinelibrary.wiley.com/doi/10.1111/ejn.13400/epdf

Page 3 of 52European Journal of Neuroscience

For Peer Review

RE: We thank the Editor for this comment. We have now replaced our previous bar charts.

7) Your figures need to be clearly labelled so readers know which is which

RE: We thank the editor for his useful comments. We have reviewed the label of each figure.

Reviewer: 1

1) The authors should carefully edit the manuscript to correct punctuation errors and a few typos.

RE: We thank the reviewer for this comment. We have now carefully gone through the manuscript and corrected any punctuation and

typographic errors that we found.

Page 4 of 52 European Journal of Neuroscience

For Peer Review

2) The kinesthetic illusion generated by high frequency vibratory stimulation consists of a false sensation of muscle contraction,

not lengthening. This is correctly reformulated in the first paragraph of the Discussion.

RE: In the revised version of the manuscript we have corrected this error. This sentence now reads: “The central nervous system

incorrectly interprets this increased firing rate of muscle spindles as if the affected muscle is contracting, which generates uncertainty in

the actual position of the limb” (page 3, lines 81-82).

3) METHODS

Experiments 1 and 2

Why was Experiment 1 reported separately, if the only difference from the first is the addition of a 3rd condition of vibratory

stimulation over the left wrist, and results of Experiment 2 incorporate results of Experiment 1? Moreover, the results of the Box

Page 5 of 52European Journal of Neuroscience

For Peer Review

and blocks test are different between Experiment 1 and Experiment 2 with respect to the comparison between the “absent

vibratory stimulation” and the “80 Hz vibration”, which is difficult to interpret (although the direction of the effect is the same).

RE: Experiment 1 and Experiment 2 were conducted separately and sequentially. These are separate experiments and not a partition of

the same data. Indeed, the order of the reporting of the experiments in the methods and results faithfully reflects the order in which the

experiments were conducted. We decided to report each experiment separately because they have been performed in different sessions

and combining them would falsely give the impression to the reader that they were conducted at the same time. The reviewer is correct to

point out a difference in the significance of the results of the box and blocks task between Expt 1 and Expt 2. In the revised version we

have included a table to collate the results of the statistical tests for ease of comparison. In addition, where there was a case where the

effect was not significant in one or more experiments we tested whether the magnitude difference between the baseline and the 80Hz

condition was significantly different between experiments. Although, both the box and block test and the reaction time test did not show

an effect in one of the experiments there were no evidence that this was due to a difference in the magnitude of the effect between

Page 6 of 52 European Journal of Neuroscience

For Peer Review

conditions. Rather it would appear that this was a result of the natural variance between participants that resulted in a null result one

time out of the four replications. This is described and discussed at the beginning of the discussion in the revised manuscript.

4) On page 5, line 2: please define the interstimulus interval (which are the stimuli separated by this interval? The reaction time

task has 1 stimulus per trial, but the indicated ISIs are very small to mean time elapsing between trials).

RE: The interstimulus interval was defined as the time between the onset of the fixation cross and the time of the GO signal. We have

changed this in the revised manuscript to make this clearer. The following sentence has been added “The time between the onset of the

fixation cross and the green GO signal was either 500ms, 750ms or 1000ms and jittered between trials so the onset of the GO signal was

not predictable” (page 5, lines 114-115).

5) The last sentence refers to the 20 Hz vibration condition, which was not part of this Experiment. This seems an editing mistake?!

Page 7 of 52European Journal of Neuroscience

For Peer Review

RE: We thank the Reviewer for spotting this mistake. We have now corrected this in Experiment 2 results section. It now reads “There

was no significant difference between the mean reaction times following 80 Hz vibration on the right wrist and 80 Hz vibration on the left

wrist (t (17) = -1.544, p = 0.1) as well as between no vibration and 80 Hz vibration on the left wrist (t (17) = -0.512, p = 0.6) (Fig.3

E&F)” (page 10, lines 273-275).

6) Experiment 3

Explain here the rationale of the 20 Hz vibratory stimulation condition.

RE: The rationale here was to try and control for a potential placebo effect of vibration. One possible explanation of the results from

Experiments 1 and 2 was that effect of vibration was just some sort of placebo effect. We have added the following: “To this end we

Page 8 of 52 European Journal of Neuroscience

For Peer Review

investigated the effect of vibration at 80 Hz and 20 Hz to control for any potential placebo effect of vibration at the wrist” (page 11, lines

282-284).

7) DISCUSSION

Can the authors speculate on why beta attenuation occurred both at the onset and at the offset of the 80 Hz vibratory

stimulation? What happened throughout the stimulation: did the beta power return to baseline, and was there any effect of

condition?

RE: The author raises an interesting point. The beta attenuation observed was greatest (and significantly different from baseline) at

vibration onset and offset. During vibration, on average the beta value was attenuated compared to baseline (see Figure 6) but this was

not significant. This attenuation only occurred for the 80 Hz vibration and not for the 20 Hz vibration as reported in the original paper

Page 9 of 52European Journal of Neuroscience

For Peer Review

and as can be seen in Figure 6. In the revised manuscript we have included a brief section to the discussion speculating on why the beta

attenuation is maximal at the onset and offset of the vibration. We have added the following to the manuscript: “Indeed, in the current

study, there was a decrease in beta oscillatory activity at the onset and offset of 80 Hz peripheral vibration, but not 20 Hz. This pattern of attenuation

appears reflective of an adaptation mechanism. Muscle spindles in general are rapidly adapting and respond to relative changes in muscle stretch

rather than sustained stretch, potentially reflecting this pattern of activity. In addition, there is evidence that the motor system adapts to a constant

vibratory stimulus and this potential top-down adaptation may be reflected in the modulation of beta power in this study. For example, there is a clear

reduction in muscle spindle activity up to 40s following the offset of vibration compared to baseline (pre-vibration) (Ribot-Ciscar et al., 1998) and

during this period participants often experience illusory aftereffects i.e. the vibrated muscle appears to move in the opposite direction to that

experienced during the vibration (Seizova-Cajic et al., 2007). This adaptation likely occurs via a central mechanism. The modulations in beta power

at the onset and offset of the vibratory stimulus may reflect the sensorimotor cortex adapting to an unexpected stimulus, which then becomes

predictable, which supports the hypothesis that this oscillatory activity reflects cortical estimates of proprioceptive uncertainty.” (page 18, lines

497-509).

Page 10 of 52 European Journal of Neuroscience

For Peer Review

8) Can the authors comment on whether they expect that the effect of high frequency vibratory stimulation in increasing

uncertainty of proprioceptive afferent input has a topographical specificity? Also, do the authors expect a difference of effect

upon voluntary, non-automatic movements as opposed to automatic motor programmes, e.g. gait? Reproducing the same effect

in the lower limbs would be of enormous interest and could support this as an adjunctive intervention for freezing of gait, which

is a failure of gait initiation.

RE: The reviewer raises several really interesting questions to which any answer would be too speculative to include in the

discussion but we can speculate here. It is likely that any updating of the proprioceptive uncertainty through vibration as performed here

would be topographic. In experiment 2 here we vibrated the left wrist and the participants then moved the right hand. In this condition

the vibration had no effect on the performance and performance was only improved when 80 Hz vibration was applied to the wrist of the

hand that subsequently performed the task. The question as to whether gait or episodes of freezing of gait would be improved is clearly of

Page 11 of 52European Journal of Neuroscience

For Peer Review

great interest and we are currently running experiments to address this question. We should know the results soon. We have added this

as a future direction in the discussion.

Reviewer: 2

Comments to the Author

1) Authors should provide more information and details about the experimental phases, because some of them are not clear. For

example, could the timeframes of the tasks be described? Did motor tasks begin as soon as the vibration stopped? This is not

clearly explained. If so, how does the reaction time task worked? Did subjects repeat this task 3 times in total or per jitter

Page 12 of 52 European Journal of Neuroscience

For Peer Review

duration? Did you find any differences between these conditions? What kind of task did subjects do with the glove? Did they only

had to tap thumb and index finger? What were the instructions? Did subjects perceived kinesthetic illusion or not?

RE: We thank the reviewer for this comment. The motor paradigm began as soon as vibration stopped in each experiment. The reaction

time was performed immediately after the vibration. The subjects repeated each task, including the reaction time 3 times. Vibration was

applied before each repetition. The fingers tapping was performed by tapping the thumb with the index finger. The details of the

experimental tasks have been added into the Methods section. In the current study we did not explicitly ask or test for the presence or

absence of a kinesthetic illusion and no subjects reported this. However, this is an interesting question and is something that we are

currently investigating. This has been highlighted in the Discussion.

2) I think that the healthy young group of subjects of Experiment 3 wasn’t the right control for the older group of PD patients.

Page 13 of 52European Journal of Neuroscience

For Peer Review

RE: The different healthy young control groups were not and were never intended to be a control group for the PD patients. In the

sequence of experiments, we first demonstrated an effect in young healthy controls and then subsequently tested whether a similar effect

was present in PD patients.

3) Author should describe in details what kind of analysis they performed for each experiment in the Data Analysis paragraph

underlining the factors, they use in every analysis. They also had to explain how the corrected means were calculated, if they

chose to use them.

Page 14 of 52 European Journal of Neuroscience

For Peer Review

RE: We have made this clearer in the revised manuscript. The mean corrected values simply remove the between subject effect by

removing the mean value across conditions for each subject. This was performed so that the reader can see in the figures the mean

within subject effects without contamination with the between subject variance. We have explained how this was performed in the revised

version.

4) How do authors explain the fact that some performances significantly improved in some experiment and does not in others? I’m

referring to, as an example, Reaction time task results in exp1 and 2. I think they have to comment this fact.

RE: In the revised manuscript we have now provide a table summary of all the results and have also performed an Omnibus analysis

where possible to test the reproducibility of the results observed. For reaction times when the different experiments were analysed with a

between group ANOVA there was a main effect of condition (no vibration and 80 Hz vibration) (F(1,68)=15.146, p<0.001) a main effect

Page 15 of 52European Journal of Neuroscience

For Peer Review

of Experiment (F(3,68)=8.898) and no significant interaction between condition and Experiment (F(3,68)=1.722, p=0.171). There were

no significant differences between the effects of reaction times in experiment 1 and experiment 2.

5) I think that figure 4 refers to experiment 4, and not experiment 3 where it is cited.

RE: We thank the reviewer for pointing our mistake. We have now corrected this.

6) Figures lacks details like titles and axis, but “raw” figures and corrected mean figures are redundant. Why did authors

indicate statistical significance over the means graphs if they performed the statistical analysis over raw values, indicated in the

main text?

Page 16 of 52 European Journal of Neuroscience

For Peer Review

RE: The raw figures and mean corrected figures are not redundant but show different aspects of the same data. The Raw values include

both between and within subject variance – but show the actual spread of values across participants, which is useful for the reader. The

mean corrected plots remove the between subject variance and is equivalent to the effects that are reported statistically. For this reason

and for completeness we would like to keep both graphical representations here.

7) I think that the discussion needs some theoretical revision, but in general authors have to explain the findings of all their

experiment more in detail and comment the literature they cite carefully.

RE: We have revised parts of the discussion and hope that that reviewer is happier with the revised version.

_____________________________________________________________________________________

2nd Editorial Decision	25/03/2018

_____________________________________________________________________________________

High frequency peripheral vibration decreases completion time on a number of motor tasks

Dear Dr. Macerollo,

Your manuscript was reviewed again by three external reviewers and the editors.  Based on these reviews, we regret to inform you that we are not able to consider your manuscript further for publication in EJN.  While one of the original reviewers was satisfied with your revision, another was not convinced by your revisions and our third reviewer questions whether the work is sufficiently neurally focused for EJN. We apologize for the long delay in getting this word to you but as you can see, we needed to deliberate carefully given these three disparate opinions. We are sorry to write with this disappointing news.

Thank you for submitting your work to EJN. We very much hope to receive papers from you in the future.

Kind regards,

John Foxe & Paul Bolam
co-Editors in Chief, EJN

REVIEWS:

Reviewer: 1

Comments to the Author
After careful revision, I feel overall satisfied of how the authors have addressed my comments and suggestions.

Reviewer: 2

Comments to the Author
Honestly, I'm not persuaded by the correction provided by authors. The description of the experimental designs remains unclear. Data seem to be reproducible the data does not seem to be reproducible and I am not persuaded by the new analysis. Some of the decisions taken do not seem justified by the results. Figures remain unclear.
Moreover, the sentence inserted in the introduction, to our knowledge, is not correct. The kinesthetic illusion generated by the vibration consist of a sensation of muscle lengthening, as previously written. Please provide a reference for the new sentence.

Reviewer: 3

Comments to the Author
I read the revision and response to both reviewers. The authors seem to have responded to most of the concerns. The manuscript is sufficiently detailed that an interested scientist could replicate the experiments.

My main concern is whether this even belongs in a neuroscience journal. The study is a curious motor-physiology phenomenon, but there is no evidence for neural mechanisms in this paper. How do we even know that these effects are mediated by the brain? Perhaps the stimulation is potentiating spinal synapses. There is EEG data but showing an enhanced EEG response for 80 compared to 20 Hz stimulation is not evidence that the improvement in RTs is cortically mediated. That finding could be due to subjects subjectively feeling the 80 Hz stimulation as being more intense.

_____________________________________________________________________________________

Authors’ Response	30/15/2018

_____________________________________________________________________________________

UCL INSTITUTE OF NEUROLOGY

QUEEN SQUARE

The National Hospital for Neurology and Neurosurgery

Queen Square

LONDON

WC1N 3BG

Sobell Department of Motor Neuroscience and Movement Disorders

23-4-2018

RE EJN-2017-08-24896.R1

Dear Editors,

Please find attached our revised manuscript based on the Reviewer’s comments on EJN-2017-08-24896.R1 and

our detailed responses to the Reviewers are in this cover letter.

The paper has been reviewed by 3 reviewers and the paper revised based on the original comments of 2

reviewers. One of the original reviewers was happy with the revisions we made and one was not. Therefore a

third reviewer was found to review the manuscript and decide whether or not we had addressed the previous

reviewer’s concerns. The third reviewer wrote "The authors seem to have responded to most of the concerns. The

manuscript is sufficiently detailed that an interested scientist could replicate the experiments". So, it would seem

that the third reviewer agreed with Reviewer 1 that the previous concerns had been addressed in the revision.

However, the paper was rejected. This seems to be based on a concern raised by the new Reviewer that the

paper was not a good fit for EJN. For reasons outlined below we believe that this main concern for Reviewer 3

is not consistent with the Aims and Scopes of EJN and we successfully appealed the rejection. This is the

resubmission of this paper which we believe now addresses all the previous concerns.

Page 1 of 37 European Journal of Neuroscience

For Peer Review

Address for correspondence: Sobell Department of Motor Neuroscience and Movement Disorders, UCL Institute of Neurology National Hospital for Neurology and Neurosurgery (Box 28) Queen Square London WC1N 3BG.

Email: j.kilner@ucl.ac.uk	www.ion.ucl.ac.uk

The Institute of Neurology promotes teaching and research of the highest quality in neurology and the neurosciences.

Below we have included the Reviewers comments (in BOLD) our replies to them (in regular font) and any

included changes to the manuscript (in italics).

Reviewer: 1

Comments to the Author

After careful revision, I feel overall satisfied of how the authors have addressed my comments and suggestions.

We thank the Reviewer for these comments and we are happy that the Reviewer now considers the paper suitable for

publication in EJN.

Reviewer: 2

Comments to the Author

Honestly, I'm not persuaded by the correction provided by authors. The description of the experimental

designs remain unclear. Data seem to be reproducible the data does not seem to be reproducible and I

am not persuaded by the new analysis. Some of the decisions taken do not seem justified by the results.

Figures remain unclear.

The Reviewer 2 is not persuaded by the new analysis and revisions however a third Reviewer who was asked to

explicitly comment on this disagrees with this assertion. See comments below. The Reviewer states that some of

the decisions taken do not seem justified by the results. All decision made on new experiments were based on

the additional controls. It is unclear which decisions the Reviewer thought we did not address. In addition, the

Reviewer states the Figures are unclear. However, these are bar graphs with SEM error bars, with the individual

data for each subject shown as well as per the EJN recommendation. Therefore, it is hard to know how to make

these clearer.

Moreover, the sentence inserted in the introduction, to our knowledge, is not correct. The kinesthetic

illusion generated by the vibration consist of a sensation of muscle lengthening, as previously written.

Please provide a reference for the new sentence.

The sentence in the introduction was edited based on previous reviewers comment that vibration causes

muscle contraction and not lengthening. However, we realise there may have been some confusion caused over

the effect of vibration on the muscle and the effect on the perception of muscle length. In the references cited in

this paragraph in the introduction an illusory sense of muscle lengthening and joint extension is generated by

vibrating the flexor muscle or tendons. However, prolonged high amplitude vibration can produce an increase

in muscle contraction referred to as the tonic vibration reflex (TVR). In the illusion experiments referenced the

perception of muscle lengthening was studied in the absence of any muscle contraction. Indeed, in the current

Page 2 of 37European Journal of Neuroscience

For Peer Review

Address for correspondence: Sobell Department of Motor Neuroscience and Movement Disorders, UCL Institute of Neurology National Hospital for Neurology and Neurosurgery (Box 28) Queen Square London WC1N 3BG.

Email: j.kilner@ucl.ac.uk	www.ion.ucl.ac.uk

The Institute of Neurology promotes teaching and research of the highest quality in neurology and the neurosciences.

study similar low amplitude high frequency vibration was employed to avoid any overt muscle contraction. The

introduction has now been corrected and made clearer. We have corrected the sentence and it now reads.

It has previously been shown that high frequency vibration of forearm muscle tendons, which selectively

activates muscle spindles (Brown et al., 1967; Burke et al., 1976; Roll et al., 1989), produces the illusion that

the arm is moving or has been displaced in the absence of any EMG activity (Goodwin et al., 1972; McCloskey,

1973; Craske, 1977). This illusion reflects the central nervous system incorrectly interpreting the increased

firing rate of muscle spindles as if the vibrated muscle is lengthening; this generates uncertainty in the actual

position of the limb. This has been demonstrated in a number of position-matching and pointing tasks all of

which show increased error, or reduced accuracy, following high-frequency peripheral vibration (Capaday and

Cooke, 1983; Inglis and Frank, 1990; Cordo et al., 1995, 2005; Tsay et al., 2016). Importantly, prolonged high

amplitude vibration (30-100Hz) can produce an increase in muscle contraction referred to as the tonic

vibration reflex (TVR). This reflects an increase in activity from afferent nerve fibres that activate

monosynaptic and polysynaptic reflex arcs (Hagbarth and Eklund, 1966). As in these previous experiments, in

the current study low amplitude vibration was employed to activate muscle spindles without any overt muscle

contraction.

Reviewer: 3

Comments to the Author

I read the revision and response to both reviewers. The authors seem to have responded to most of the

concerns. The manuscript is sufficiently detailed that an interested scientist could replicate the

experiments.

We thank the Reviewer for these comments and that the Reviewer agrees with Reviewer 1 that the revised

manuscript addresses the previous concerns and that the methodologies are detailed to be understood and

replicated.

My main concern is whether this even belongs in a neuroscience journal. The study is a curious motor

physiology phenomenon, but there is no evidence for neural mechanisms in this paper. How do we even

know that these effects are mediated by the brain? Perhaps the stimulation is potentiating spinal

synapses. There is EEG data, but showing an enhanced EEG response for 80 compared to 20 Hz

stimulation is not evidence that the improvement in RTs is cortically mediated. That finding could be due

to subjects subjectively feeling the 80 Hz stimulation as being more intense.

Page 3 of 37 European Journal of Neuroscience

For Peer Review

Address for correspondence: Sobell Department of Motor Neuroscience and Movement Disorders, UCL Institute of Neurology National Hospital for Neurology and Neurosurgery (Box 28) Queen Square London WC1N 3BG.

Email: j.kilner@ucl.ac.uk	www.ion.ucl.ac.uk

The Institute of Neurology promotes teaching and research of the highest quality in neurology and the neurosciences.

The paper describes the results of 5 behavioural experiments, and one EEG study. The comment that our work

is not suitable for EJN is particularly surprising mainly because the neural processes in the spine are considered

neuroscience; neuroscience is not exclusively about the brain. It is not my understanding that research articles

published in EJN should be exclusively about the brain, at the exclusion of the spine or other parts of the CNS.

Indeed, the information on the EJN website is not consistent with the Reviewers comments. In the Aims and

Scopes section it is written "EJN publishes original research articles and reviews in the broad fields of

molecular, cellular, systems, behavioral, cognitive and computational neurosciences." The statement that EJN

accepts behavioural articles seems at odds with Reviewer 3’s main concern with the paper. Based on this we

think that Reviewer 3 is mistaken in their understanding of the aims of scopes and we believe our work is

entirely suitable for publication in EJN.
